# In eigener Sache

**DOI:** 10.1007/s11615-020-00272-0

**Published:** 2020-09-02

**Authors:** Eric Linhart

**Affiliations:** Institut für Politikwissenschaft, Technische Universität Chemnitz, Thüringer Weg 9, 09126 Chemnitz, Deutschland

Liebe Leserinnen und Leser,

mit dem 61. Jahrgang standen und stehen für die PVS sowohl personelle als auch inhaltliche Veränderungen an, über die ich Sie in diesem Editorial gerne informieren möchte. Bereits zum Jahreswechsel 2019/2020 ist die Hauptredaktion der PVS von Darmstadt nach Chemnitz gewechselt, und die redaktionelle Leitung der Fachzeitschrift wurde von Michèle Knodt auf mich übertragen. Damit verbunden ist auch Katharina Kleinschnitger als Managing Editor ausgeschieden; ihr Amt hat Niclas Hüttemann übernommen. Während der Wechsel offiziell zum Jahresbeginn vollzogen wurde, war das Darmstädter Team noch für die Hefte 1 und 2 des 61. Jahrgangs allein- bzw. mitverantwortlich. Spätestens jetzt, wo mit Heft 3 die Stabübergabe nach Chemnitz vollständig abgeschlossen ist, ist es an der Zeit, Michèle Knodt und Katharina Kleinschnitger auf das Herzlichste für ihre geleistete Arbeit in den vergangenen Jahren zu danken. Gemeinsam mit den anderen Redaktionsmitgliedern haben sie maßgeblich zur Weiterentwicklung und Profilierung der PVS beigetragen. Am Ende ihrer Amtszeiten haben sie für eine Übergabe gesorgt, wie sie reibungsloser nicht hätte sein können.

Zum Jahresbeginn ist auch das für „Internationale Beziehungen“ zuständige Redaktionsmitglied, Mathias Albert (Universität Bielefeld) ausgeschieden. Auch ihm sei für die geleistete Arbeit herzlich gedankt! Der von ihm verantwortete Teilbereich wird seit Mai von Margit Bussmann (Universität Greifswald) betreut. Weiterhin zuständig für die Bereiche „Empirische Sozialforschung und Methodenlehre“ sowie „Politische Soziologie“ ist Kai-Uwe Schnapp (Universität Hamburg). Um die „Policy-Analyse“ kümmert sich seit vergangenem Jahr Christian Adam (Ludwig-Maximilians-Universität München); der Bereich „Analyse und Vergleich politischer Systeme“ wird in Chemnitz durch mich abgedeckt. Für die „Politische Theorie und Ideengeschichte“ ist weiterhin Michael Haus (Ruprecht-Karls-Universität Heidelberg) zuständig. Hierbei wird er von Dirk Jörke (TU Darmstadt) unterstützt, dessen Hauptaufgabe seit 2019 die Betreuung des Literaturbereichs der PVS ist.

Während die personellen Veränderungen bereits vollzogen sind, stehen die inhaltlichen Neuerungen ab Heft 4 an. Die Redaktion hat sich auf ihrer jüngsten Redaktionskonferenz – in Zeiten von Covid-19 selbstverständlich virtuell – unter anderem mit den unterschiedlichen Formaten der PVS befasst. Hierbei wurden insbesondere das *Forum* und der *Kommentar* intensiv diskutiert. Beide Formate haben in der jüngeren Vergangenheit nach Meinung der Redaktion eine Entwicklung vollzogen, die es zumindest partiell schwierig macht, sie klar voneinander abzugrenzen. Je nach Ausrichtung des Forumsbeitrags oder Kommentars verschwimmen auch die Grenzen zur *Abhandlung*. Die Redaktion hat daher die Einstellung dieser beiden Formate beschlossen. Da es uns gleichzeitig wichtig ist, in der PVS auch Raum für kontroverse Debatten aktueller politischer und politikwissenschaftlicher Entwicklungen zu geben, führen wir hierfür die Kategorie *Debatte* ein. Streng wissenschaftlich ausgerichtete Beiträge, die kürzer und gegebenenfalls auch thematisch enger als Abhandlungen sind, können künftig als *Research Note* in der PVS publiziert werden. In Research Notes ist auch die kritische Auseinandersetzung mit zuvor in der PVS erschienenen Beiträgen möglich. Schließlich werten wir in Zukunft *Literaturberichte* auf, indem wir sie wie Abhandlungen und Research Notes einem doppelt-blinden Begutachtungsverfahren unterziehen. Sie sollen noch stärker als bisher einen umfassenden Überblick des Forschungsstands zu einem speziellen politikwissenschaftlichen Thema geben. In Abgrenzung zum bisherigen Format nennen wir die aufgewertete Kategorie zukünftig *Literaturübersicht*. Erhalten bleiben neben der klassischen *Abhandlung* auch *Rezensionen* und *Sammelrezensionen*. Weiterführende Hinweise zu allen Formaten sind auf der Homepage der PVS zu finden.

Wir hoffen, mit dieser Neuausrichtung der Formate in der PVS sowohl für unsere Autorinnen und Autoren als auch für unsere Leserinnen und Leser eine klarer nachvollziehbare Struktur geschaffen zu haben. Zudem freuen wir uns, wenn unsere Autorinnen und Autoren die zusätzlichen Möglichkeiten, die unsere neuen Formate bieten, rege nutzen werden. Wir bitten um Verständnis dafür, dass Einreichungen in den auslaufenden Kategorien ab jetzt nicht mehr möglich sind. Einreichungen, die bereits erfolgt sind, werden selbstverständlich zu einem – hoffentlich erfreulichen – Abschluss gebracht, sodass Sie auch in den kommenden Heften der PVS noch vereinzelt Forumsbeiträge, Kommentare und Literaturberichte finden werden.

Eric Linhart

